# Effects of Seasonal Upwelling on Inorganic and Organic Matter Dynamics in the Water Column of Eastern Pacific Coral Reefs

**DOI:** 10.1371/journal.pone.0142681

**Published:** 2015-11-11

**Authors:** Ines Stuhldreier, Celeste Sánchez-Noguera, Tim Rixen, Jorge Cortés, Alvaro Morales, Christian Wild

**Affiliations:** 1 Leibniz Center for Tropical Marine Ecology, Bremen, Germany; 2 Faculty of Biology and Chemistry, University of Bremen, Bremen, Germany; 3 Centro de Investigación en Ciencias del Mar y Limnología, Universidad de Costa Rica, San Pedro, San José, Costa Rica; University of Vigo, SPAIN

## Abstract

The Gulf of Papagayo at the northern Pacific coast of Costa Rica experiences pronounced seasonal changes in water parameters caused by wind-driven coastal upwelling. While remote sensing and open water sampling already described the physical nature of this upwelling, the spatial and temporal effects on key parameters and processes in the water column have not been investigated yet, although being highly relevant for coral reef functioning. The present study investigated a range of water parameters on two coral reefs with different exposure to upwelling (Matapalo and Bajo Rojo) in a weekly to monthly resolution over one year (May 2013 to April 2014). Based on air temperature, wind speed and water temperature, three time clusters were defined: a) May to November 2013 without upwelling, b) December 2013 to April 2014 with moderate upwelling, punctuated by c) extreme upwelling events in February, March and April 2014. During upwelling peaks, water temperatures decreased by 7°C (Matapalo) and 9°C (Bajo Rojo) to minima of 20.1 and 15.3°C respectively, while phosphate, ammonia and nitrate concentrations increased 3 to 15-fold to maxima of 1.3 μmol PO_4_^3-^ L^-1^, 3.0 μmol NH_4_^+^ L^-1^ and 9.7 μmol NO_3_^-^ L^-1^. This increased availability of nutrients triggered several successive phytoplankton blooms as indicated by 3- (Matapalo) and 6-fold (Bajo Rojo) increases in chlorophyll *a* concentrations. Particulate organic carbon and nitrogen (POC and PON) increased by 40 and 70% respectively from February to April 2014. Dissolved organic carbon (DOC) increased by 70% in December and stayed elevated for at least 4 months, indicating high organic matter release by primary producers. Such strong cascading effects of upwelling on organic matter dynamics on coral reefs have not been reported previously, although likely impacting many reefs in comparable upwelling systems.

## Introduction

Coral reefs require warm, sunlit, clear, oligotrophic and carbonate-supersaturated conditions for optimal growth [1]. Sedimentation and turbidity, nutrient availability, amounts and types of organic matter in the water, contaminants, salinity, temperature and alkalinity can all strongly influence the productivity, resilience and function of coral reef ecosystems [2]. While coral reef environments are characterized by a high degree of stability [3], higher instability of conditions is projected for future coral reefs, as global stressors such as ocean warming and acidification along with local pressures such as eutrophication and pollution from land are increasing [4,5]. The potential of coral reefs to adapt to these environmental changes is still under debate [6,7]. Some reefs existing in areas exposed to high natural variations in water quality may serve as natural laboratories to study the effects of changes in environmental parameters on coral reef functioning. Reefs along the Pacific coast of Mesoamerica for instance are exposed to highly dynamic water conditions in space and time. The Eastern Pacific Warm Pool with sea surface temperatures above 27°C is interrupted by seasonal coastal upwelling zones in the gulfs of Tehuantepec (Mexico), Papagayo (Costa Rica—Nicaragua), and Panama (Panamá) [8,9]. These upwelling systems are caused by narrow wind jets blowing from land to sea during the northern hemisphere winter, when high pressure systems in the Caribbean promote strong winds that are canalized through topographical gaps in the volcanic mountain range of Mesoamerica [10,11]. In response to wind forcing, water currents in the Pacific displace superficial water away from the coast, causing an uplift of the shallow thermocline [12]. In combination with intense vertical mixing by high wind speeds, this phenomenon brings water with low temperature, low pH and high concentrations of nutrients to the surface between November and April [13]. In the Gulf of Papagayo, temperatures of 10°C below the annual mean and down to < 15°C have been measured during upwelling months [11,14,15], and drops in pH from 8.01 to 7.86 units within 30 minutes were recorded during upwelling events [16]. Nutrient concentrations up to 15 μmol L^-1^ nitrate and 1.3 μmol L^-1^ phosphate were measured during upwelling periods in the Gulfs of Papagayo and Panama [12,17].

Low temperatures, high nutrient concentrations and the fast changes in these key water parameters are unfavorable for coral growth. However, coral communities and reefs occur along the northern Pacific coast of Costa Rica despite the occurrence of seasonal upwelling [18]. So far it is known that the northern Pacific coast of Costa Rica experiences strong seasonal variations in oceanographic parameters, but this information derives from remote sensing or sampling of the open water column. The spatial and temporal effects of upwelling on organic parameters and processes in the water column which are relevant for coral reef functioning have not been investigated yet. Therefore, this study monitored temporal and spatial variability of temperature, salinity, pH, dissolved oxygen, inorganic nutrient and chlorophyll *a* concentrations as well as particulate and dissolved organic matter. Monitoring was conducted on two coral reefs differently exposed to upwelling in a weekly to monthly temporal resolution over a period of 12 months. The goal was to describe how long and to what extent key water column parameters on coral reefs in the Gulf of Papagayo are influenced by seasonal upwelling. In contrast to oceanographic data from previous studies, the data presented here illustrate fine-scale trends and processes in reef waters.

## Methods

### Study sites

**Fig 1:**
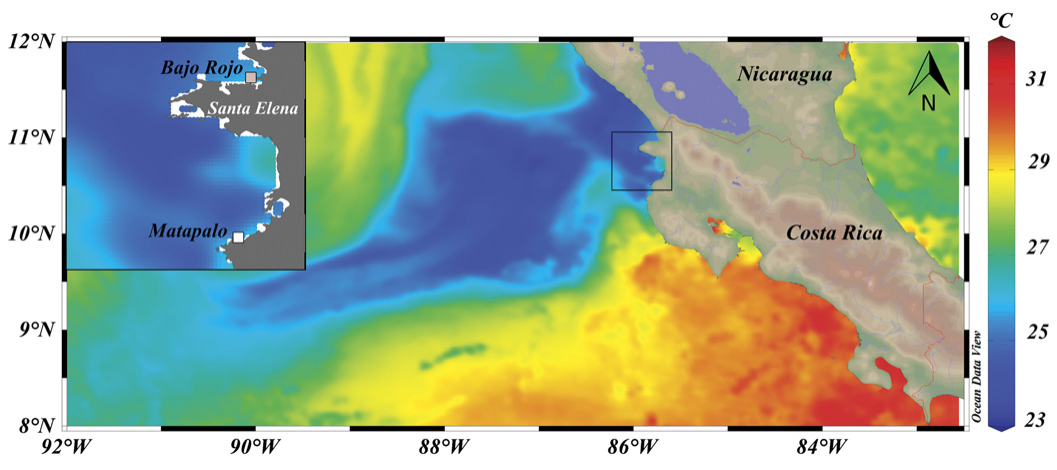
Upwelling event at the Pacific coast of Costa Rica on 17 February 2014. Color scale in the water indicates sea surface temperature (SST). Shading on land indicates altitude; note the depression in the volcanic mountain range at Lake Nicaragua that enables Trade Wind crossing from the east. The inset shows the locations of study sites Matapalo (10°32’21”N, 85°45’59”W) and Bajo Rojo (10°57’26”N, 85°43’59”W). SST data were derived from a daily, global 1-km SST data set (GHRSST, Level 4, G1SST) produced by the JPL Regional Ocean Modeling System group [19], available at http://ourocean.jpl.nasa.gov/SST/ (accessed 09.12.2014). Data were visualized with the software Ocean Data View (Schlitzer, R., Ocean Data View, http://odv.awi.de, 2013).

Water conditions were monitored at two reef sites in the Gulf of Papagayo, situated in 46.3 km air-line distance to each other (Fig 1). Matapalo reef is dominated by the branching coral *Pocillopora* spp. and extends around 1 km along the northern coast of the Nicoya Peninsula, with alternating patches of dead and living carbonate structure in 3–8 m water depth. An area of around 600 m^2^ with relatively high live coral cover compared to the surrounding area was visited weekly. North of the Santa Elena Peninsula the upwelling is stronger, because the trade wind flow from the Caribbean during the northern hemisphere winter is not blocked by the volcanic mountain range of Central America (Fig 1). The study site Bajo Rojo is a small rocky outcrop 2 km off the coast, where a reef dominated by *Pavona gigantea* stretches around 70 m along the base of the rock in 7–11 m water depth. This site was visited monthly. Data from Matapalo are discussed in detail, while data from Bajo Rojo are described in relation to Matapalo. Necessary field permits were granted by the National System of Conservation Areas (SINAC) of Costa Rica.

### Seasonal periods

**Fig 2:**
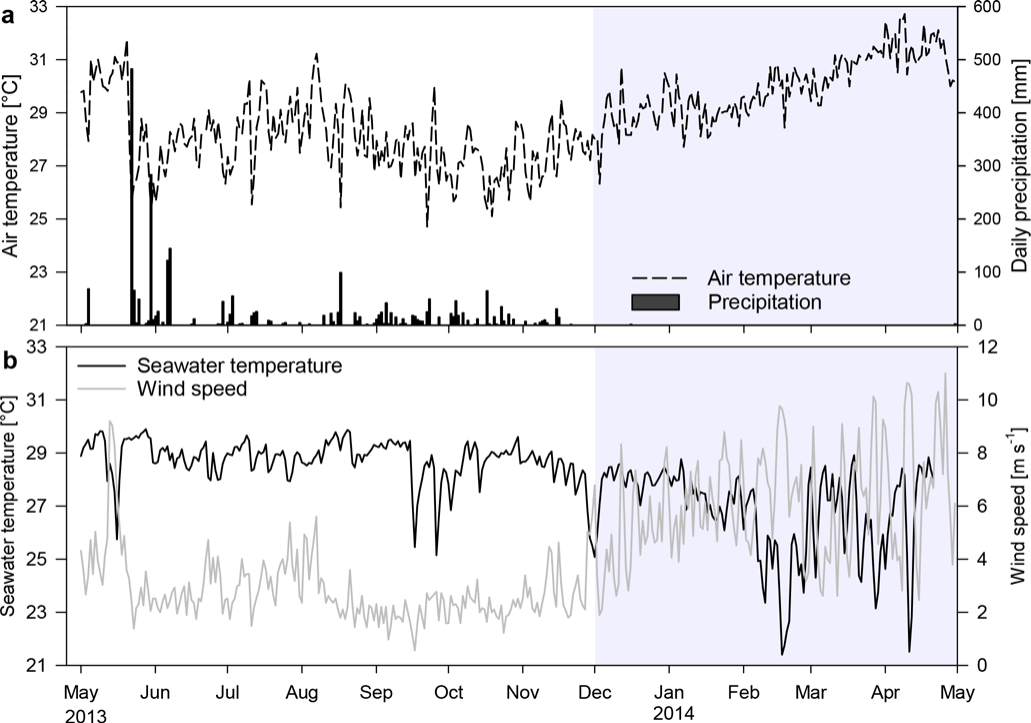
Changes in meteorological parameters and seawater temperature in 5 m depth at Matapalo over 12 months. (a) Mean daily air temperature [°C] and daily precipitation [mm]. (b) Mean daily seawater temperature [°C] and mean daily wind speed [m s^-1^]. Shaded area = upwelling period.

The 12 month study period from 01 May 2013 to 21 April 2014 encompassed the rainy season from May to November, and the dry trade-wind season from December to April. The transitions between these seasons are not well defined, and periods may vary between years. For this study, we defined time periods using a multivariate clustering routine in PRIMER 6 based on similarity in air temperature, wind speed and water temperature between 15 April 2013 and 21 April 2014. Meteorological data (Fig 2) originated from the Daniel Oduber Quirós International Airport station (10°35’35”N, 85°32’44”W, 80 m above sea level), 25 km northeast of Matapalo and 45 km southeast of Bajo Rojo. Daily averages of air temperature and wind speed were obtained from http://www.ncdc.noaa.gov/cdo-web/datasets (accessed July 2014). Water temperature was measured in 5 m depth at Matapalo in 5–30 min intervals (see below) and calculated to daily averages. The cluster analysis (resemblance based on Euclidean distance, complete linkage) resulted in three groups: a) non-upwelling period (noUPW) including 215 days mainly from May to November 2013, b) upwelling period (UPW) including most days in April 2013, some days over the year and most days from December 2013 to April 2014 and c) extreme upwelling (extUPW) including 15 days in February, March and April 2014. Based on this analysis, the days of weekly/monthly sampling were assigned to their respective cluster (n_noUPW_ = 34, n_UPW_ = 20, n_extUPW_ = 5) which was later used as a factor to determine the differences in environmental parameters between seasons.

### Monitored parameters

Water temperature was recorded continuously at both study sites with HOBO^®^ Pendant Temperature Data loggers deployed directly above the reef substrate (5 min intervals between February and April, 10–15 min between December and January, 30 min during non-upwelling) and was calculated to daily averages. Due to loss and malfunction of loggers, there are no data available for Bajo Rojo from 12 September 2013 to 12 November 2013 and from 20 November 2013 to 10 December 2013.

Temperature, salinity, dissolved oxygen (DO) concentration and pH were recorded in 4 min intervals with Manta 2 Water Quality Multiprobes (Eureka Environmental Engineering) by placing the multisensors directly on the reef substrate (distance sensors to reef structure around 10 cm) for several hours between 9:00 and 16:00 during weekly/monthly observations. Daily averages were calculated from all available data points and variability over the measurement period is indicated by standard errors. Between December 2013 and April 2014, 11 temporal profiles over 7 days were recorded at Matapalo to correlate water parameters to each other and to determine daily ranges of DO concentrations and pH. Sensors were calibrated approximately every four weeks via 2-point calibration using A. dest. and 0.5 M KCl solution (four-electrode conductivity sensor), 1-point calibration using air-saturated water (optical DO sensor) and 2-point-calibration using pH 4 and 7 solutions (pH electrode), according to the manufacturer’s recommendations. The pH sensor readings were corrected for small logger specific differences and for temporal drifts in 7-day profiles, as readings dropped by 0.2 after 2 days of constant measurement. The temporal drift indicated that pH readings were too high during the weekly short-term deployment of sensors. Therefore, all sensor measured pH values were adjusted to pH values calculated from the discrete measurement of total alkalinity and dissolved inorganic carbon in seawater samples from the same study site (procedures according to [20]; calculated correction factor based on data available for 50% of the sampling days: pH_corr_ = pH/1.038).

### Water analyses

Water for the determination of dissolved organic carbon (DOC) and inorganic nutrient concentrations was sampled in triplicates from directly above the reef substrate (10–20 cm distance) in seawater washed 500 mL glass jars closed with glass lids. Directly after the dive, samples were filtered and stored cool for transportation. All syringes and containers were pre-washed twice with the respective sample, and powder-free gloves were used to avoid contamination. For DOC analysis, samples were filtered through pre-combusted glass microfiber filters (VWR, 25 mm, particle retention 0.7 μm) in polycarbonate syringe-filter-holders into acid-washed 30 mL high-density polyethylene (HDPE) wide-neck bottles and frozen at -20°C within 3 h after sampling. For analysis, samples were defrosted, acidified with 28 μL 33% HCl per 30 mL sample to reach pH ≤ 2, and analyzed in a Shimadzu TOC-VCPH + ASI-V elemental analyzer. Samples were sparged with ultra-pure carrier grade air for 10 min to drive off inorganic carbon and analyzed for Non Purgeable Organic Carbon (NPOC) using high temperature combustion (680°C) and detection of CO_2_ by a non-dispersive infrared detector conforming to U.S. EPA Method 415.1 [21,22] (number of injections: 5; limit of detection (LOD) = 8 μmol L^-1^; Reference material: zero sample (Milli-Q) after each sample and Hansell’s consensus reference material (42.5 μmol L^-1^) every 10 samples [23]). Samples for inorganic nutrient concentrations were filtered through disposable syringe filters (pore size 0.45 μm) into darkened, acid-washed 15 mL glass (for ammonia NH_4_^+^ and phosphate PO_4_^3-^) or 50 mL polypropylene containers (for nitrate NO_3_^-^ and nitrite NO_2_^-^). NH_4_^+^ was determined fluorimetrically within 24 h after sampling (Trilogy^®^ Laboratory Fluorometer/Photometer, Turner Designs) after overnight incubation with OPA (orthophthaldialdehyde)-solution in the dark (LOD = 0.023 μmol L^-1^) [24,25]. Spectrophotometric determinations of PO_4_^3-^ were conducted with the same device (LOD = 0.033 μmol L^-1^, path length = 1 cm) [26]. Samples in polypropylene containers were kept dark and frozen until the end of the study period and were analyzed spectrophotometrically for NO_3_^-^ and NO_2_^-^ concentrations (Thermo Scientific UV Evolution 201^®^) before and after reduction of NO_3_^-^ to NO_2_^-^ with vanadium (III) (LOD_(NO2_-_)_ = 0.151 μmol L^-1^, LOD_(NOx)_ = 0.162 μmol L^-1^, path length = 1 cm) [27].

Samples for the determination of chlorophyll *a* (chl *a*) and particulate organic matter (POM) concentrations were taken in triplicate in 3.8 L pre-washed plastic bottles from around 20 cm below the sea surface over the reef at the end of weekly/monthly visits. Within 3 h after sampling, subsamples of each container (1 L for chl *a*, 2 L for POM after gentle agitation of containers) were filtered onto VWR glass microfiber filters (47 mm, particle retention 1.6 μm) with an electric vacuum pump (max. pressure < 200 mbar). Due to restricted particle retention in the picoplankton size range (0.2–2 μm), values should be considered as conservative estimates of chl *a* and POM. Directly after filtration, chl *a* filters were homogenized in 7 mL 90% acetone with a glass rod, and the filter slurry was incubated overnight at 4°C. Samples were centrifuged for 10 min at 805 g before an aliquot of the supernatant was transferred to a glass cuvette. Fluorescence was measured (Trilogy^®^ Laboratory Fluorometer/Photometer) before and after acidification to 0.003 N HCl with 0.1 N HCl for 90 seconds. Procedure and calculations were carried out according to U.S. EPA Method 445.0 [28]. The pre-combusted filters with POM were stored in combusted tinfoil and frozen at -20°C until the end of the study period. Filters were dried for 24 h at 40°C for transport and again for 24 h at 40°C just before analysis. Dried filters were analyzed for total carbon (C), nitrogen (N) and organic carbon (C_org_) content in a CHN elemental analyzer (Eurovector Euro EA 3000). A quarter of the filter was used for i) the determination of C and N in tin-cups and ii) C_org_ in silver-cups after acidification with 200 μL 1 N HCl. Precision of analyses was calculated from low soil standard (OAC 187560) measured after every 5 samples (C: ± 0.032%, N: ± 0.004%, C_org_: ± 0.046%). As C_org_ was almost identical to C over the entire study period, only values for particulate organic carbon (POC) and particulate organic nitrogen (PON) in the water column are shown in the following.

### Statistical analysis

If not stated otherwise, data are always displayed as means ± standard error (SE). Statistical analyses were performed with weekly/monthly data points of environmental variables resulting in n = 59 independent samples for each of the 10 variables. Analyses of chl *a*, DOC, POC and PON did not start before July 2013. Missing values (n = 12 for chl *a*, n = 15 for DOC, n = 11 for POC and PON, n = 6 for NO_3_^-^) resulted in lower sample sizes for respective individual parameters and for multivariate analyses (noUPW = 24, UPW = 15, extUPW = 5).

Multivariate analyses were performed using the software PRIMER-E version 6 with PERMANOVA+ add-on [29,30]. Prior to analyses, temperature, PO_4_^3-^, NH_4_^+^ NO_3_^-^ and chl *a* were log transformed to reduce the skewness of data distribution (evaluated via draftman plots) and thereby ensure the homogeneity of dispersion (tested with PERMDISP prior to analyses; all p > 0.05). The parameter PON was removed from the dataset as it strongly correlated (r > 0.9) with POC, which may compromise the analyses (all other correlations r < 0.7). Environmental variables were then normalized and resemblance matrices were calculated based on Euclidean similarity. The effects of Season (nonUPW, UPW, extUPW) and Site (Matapalo, Bajo Rojo) were examined on i) all water parameters, ii) physicochemical water parameters (temperature, salinity, pH, oxygen), iii) inorganic nutrients (PO_4_^3-^, NH_4_^+^, NO_3_^-^), and iv) organic parameters (chl *a*, DOC, POC) by permutation multivariate analyses of variance (PERMANOVA), followed by pair-wise comparisons among seasons by additional post hoc PERMANOVA routines (using permutation of residuals under a reduced model and sum of squares Type III, number of permutations n = 999). Additionally, univariate ANOVAs with subsequent Tukey Tests were performed for each environmental variable and factor separately in R 3.1.1 (function aov and TukeyHSD). Inorganic nutrients and organic parameters were log(x+1) (NO_3_^-^) or log (other parameters) transformed prior to analysis to ensure normal distribution of data (graphical evaluation based on residual quantile-quantile (QQ) plots).

## Results

### Effects of Season and Site

Multivariate analyses showed significant differences between seasons (non-upwelling, upwelling, extreme upwelling) for i) all water parameters, ii) physicochemical water parameters (temperature, salinity, pH, oxygen), iii) inorganic nutrients (PO_4_^3-^, NH_4_^+^, NO_3_^-^), and iv) organic parameters (chl *a*, DOC, POC). The effects of Season and all pairwise tests were p < 0.01 except the comparison of noUPW to UPW in inorganic nutrients (*p* = 0.251) and UPW to extUPW in organic parameters (*p* = 0.185). Subsequent univariate analysis confirmed seasonal differences in all parameters except dissolved oxygen, NH_4_^+^, chl *a* and POC. The differences in single parameters were mostly detected between extUPW and noUPW (all except DOC and POC), but also between extUPW and UPW (temperature, pH, PO_4_^3-^, NO_3_^-^), and between noUPW and UPW (DOC). Differences between sites (Matapalo, Bajo Rojo) were only significant for i) all water parameters and ii) physicochemical water parameters. Univariate analyses revealed site specific differences in temperature, salinity, pH, oxygen, PO_4_^3-^ and chl *a*.

### Temporal variability of water parameters at Matapalo Reef

From May 2013 to April 2014, Matapalo reef experienced high variability in temperature (20.1–30.6°C), salinity (30.6–34.3), pH (7.83–8.38), oxygen (4.2–9.1 mg L^-1^), phosphate (0.04–1.30 μmol L^-1^), ammonia (0.22–2.53 μmol L^-1^), nitrate (below detection limit– 6.74 μmol L^-1^), chlorophyll *a* (0.11–2.22 μg L^-1^), POC (95.3–726.9 μg L^-1^), PON (13.1–118.8 μg L^-1^) and DOC (77.5–293.6 μmol L^-1^). Average values for each seasonal period are displayed in Table 1.

**Table 1:** Mean environmental parameters (± SE) at Matapalo Reef in 5 m water depth during the three main seasonal periods.

	non-upwelling	upwelling	extreme upwelling
Water column Matapalo	nUPW	UPW	extUPW
Temperature [°C]	28.56 ± 0.18 (26)	28.02 ± 0.25 (17)	23.59 ± 0.72 (4)
pH	8.03 ± 0.01 (26)	8.08 ± 0.01 (17)	8.28 ± 0.01 (4)
Salinity	32.56 ± 0.18 (26)	32.94 ± 0.26 (17)	33.80 ± 0.14 (4)
Dissolved O_2_ [mg L^-1^]	7.07 ± 0.14 (26)	7.19 ± 0.08 (17)	6.27 ± 0.48 (4)
Phosphate [μM]	0.28 ± 0.02 (26)	0.21 ± 0.03 (17)	0.77 ± 0.20 (4)
Ammonia [μM]	0.60 ± 0.05 (26)	0.66 ± 0.10 (17)	1.39 ± 0.41 (4)
Nitrate [μM]	0.50 ± 0.06 (25)	0.36 ± 0.11 (14)	3.55 ± 1.21 (4)
Chlorophyll *a* [μg L^-1^]	0.59 ± 0.08 (19)	0.69 ± 0.12 (14)	1.20 ± 0.50 (4)
Particulate N [μg L^-1^]	27.22 ± 2.54 (20)	43.33 ± 8.86 (14)	57.48 ± 12.26 (4)
Particulate organic C [μg L^-1^]	226.88 ± 28.53 (20)	300.12 ± 52.59 (14)	327.68 ± 51.62 (4)
Dissolved organic C [μM]	110.98 ± 7.25 (18)	195.72 ± 12.69 (13)	137.31 ± 11.33 (4)

Variables were measured constantly (water temperature; daily averages), weekly for several hours (pH, salinity, dissolved O_2_; sampling day average) or weekly in triplicate (nutrients and particulate as well as dissolved organic matter; sampling day average). The number of replicates for each parameter and season is displayed in brackets.

#### Physicochemical parameters

**Fig 3:**
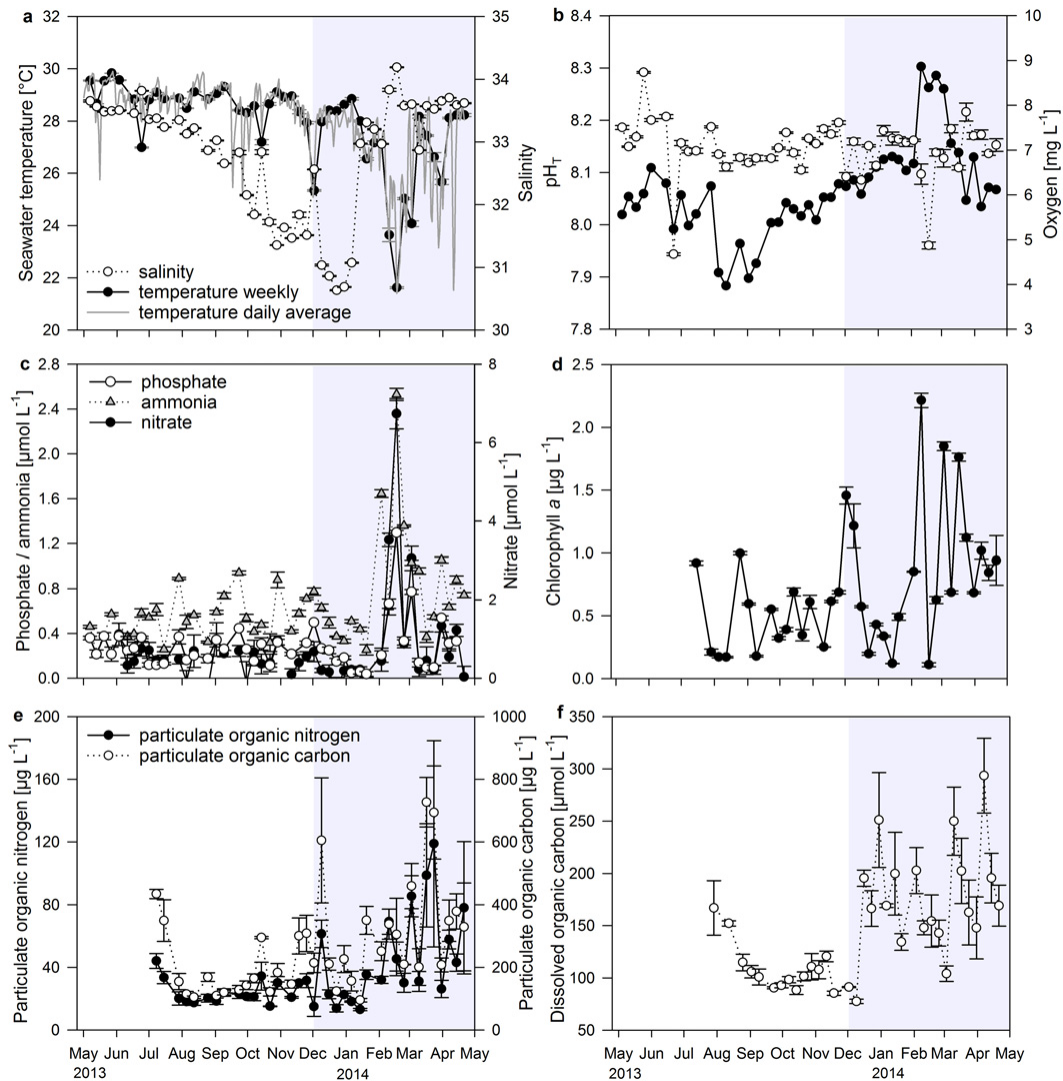
Changes in environmental parameters at Matapalo in a weekly resolution over 12 months. (a) Seawater temperature (weekly data points measured with MANTA loggers and daily averages calculated from continuous HOBO logger data) and salinity; (b) pH value (total scale) and concentration of dissolved oxygen; (c) concentrations of inorganic nutrients phosphate, ammonia and nitrate; (d) concentration of chlorophyll *a*; (e) concentrations of particulate organic nitrogen and carbon; (f) concentration of dissolved organic carbon. Error bars indicate ± SE. Shaded area = upwelling period.

The non-upwelling season from May to November 2013 was characterized by high and stable sea surface temperatures. However, pronounced but short drops to 24.4°C were observed in May and September 2013, lasting 2–5 days. These drops in temperature followed days with unusually high wind speeds, except two cold water intrusions in September 2013, when no elevated wind speeds were recorded (Fig 2B). After a first upwelling event in December 2013, temperatures returned to around 28°C before dropping down to 23.4°C in February 2014. Over the following three months, mean daily seawater temperatures dropped repeatedly by 2.2 to 6.8°C to minimum daily averages of 21.4°C for 4–6 days, after which temperatures returned to 26–29°C. Salinity was negatively correlated to seawater temperature during upwelling season (*r* = -0.59, *n* = 4730, *p* < 0.001) but also experienced a climatic pattern independent of upwelling. Lowest salinities of 30.6 occurred in December 2013, but quickly increased to ≥ 33.0 with the beginning of dry season. Maximum salinities of 34.2 in February were associated with upwelling events (Fig 3A). During weekly samplings, the reef water was usually well oxygenated (7.21 ± 0.01 mg L^-1^; 100–120% saturation). Exceptionally low DO concentrations of 4.6 mg O_2_ L^-1^ on 24 June 2013 and 17 February 2014 were associated with upwelling of cold water (Fig 3A and 3B), but average concentrations did not decrease significantly during upwelling season. Weekly pH values were significantly higher in upwelling season compared to non-upwelling and highest in February 2014 when upwelling was strongest (Fig 3B). From 7-day profiles it was visible that DO concentrations and pH experienced strong daily oscillations with lowest values during early morning hours and highest values during midday. DO concentrations varied between 4.51 ± 0.08 and 7.92 ± 0.08 mg L^-1^ O_2_ (*n* = 73), and pH between 7.87 ± 0.01 and 8.02 ± 0.01 units (*n* = 47) within 24 hours. Although weekly DO concentrations were not decreased during upwelling, minimum oxygen values in the early morning were significantly decreased during extreme upwelling between February and April (4.37 ± 0.11 mg O_2_ L^-1^) compared to values recorded in December and January (4.73 ± 0.08 mg O_2_ L^-1^; t = 2.26, df = 71, *p* = 0.027).

#### Inorganic nutrients

Inorganic nutrient concentrations were low and stable during non-upwelling and upwelling season despite occasional drops in temperature. They increased significantly by 260% (PO_4_^3-^), 110% (NH_4_^+^) and 880% (NO_3_^-^) during extreme upwelling compared to upwelling, reaching maximum values of 1.3 μmol PO_4_^3-^ L^-1^, 2.5 μmol NH_4_^+^ L^-1^ and 6.7 μmol NO_3_^-^ L^-1^ during the strongest recorded upwelling event on 17 February 2014 (Fig 3C). Nitrite concentrations stayed very low during the whole year and are therefore not shown in the following.

#### Organic parameters

Chl *a* concentrations doubled during extreme upwelling compared to upwelling and non-upwelling season. Concentrations peaked once in December 2013 and several times between February and April 2014, reaching maximum values of 2.2 μg L^-1^ (Fig 3D). While inorganic nutrient concentrations decreased after March, chl *a* stayed elevated at around 1 μg L^-1^ until the end of April. POC and PON in the water column increased temporary after a short upwelling event in December, and more persistent in March and April 2014, resulting in 30% (POC) and 60% (PON) higher values in upwelling compared to non-upwelling season (Fig 3E). While inorganic nitrogen, represented by NO_3_^-^ and NH_4_^+^, decreased after March, organic nitrogen in the form of PON stayed elevated for at least 3 more weeks, until the end of the sampling period. Between February and April 2014, the reef experienced approximately 39 and 46% of the annual POC and PON production. DOC increased by 70% two weeks after the first short upwelling event in December 2013 and stayed elevated for the following 4 month. Peaks in DOC followed elevations in chl *a*, POC and PON concentrations with a delay of 2–4 weeks (Fig 3F).

### Comparison between sites Matapalo and Bajo Rojo

Water parameters at Bajo Rojo followed similar seasonal patterns than those at Matapalo (Table 2).

**Table 2:** Mean environmental parameters (± SE) at Bajo Rojo Reef in 10 m water depth during the three main seasonal periods.

	non-upwelling	upwelling	extreme upwelling
Water column Bajo Rojo	nUPW	UPW	extUPW
Temperature [°C]	27.3 ± 0.1 (134)	23.5 ± 0.2 (124)	18.8 ± 0.4 (15)
pH	7.96 ± 0.02 (8)	7.96 ± 0.08 (3)	8.03 (1)
Salinity	33.24 ± 0.22 (8)	33.74 ± 0.31 (3)	34.70 (1)
Dissolved O_2_ [mg L^-1^]	5.77 ± 0.35 (8)	5.88 ± 0.56 (3)	5.61 (1)
Phosphate [μM]	0.38 ± 0.05 (8)	0.48 ± 0.04 (3)	1.21 (1)
Ammonia [μM]	0.57 ± 0.16 (8)	1.36 ± 0.80 (3)	0.47 (1)
Nitrate [μM]	0.50 ± 0.23 (7)	4.50 ± 3.13 (2)	9.71 (1)
Chlorophyll *a* [μg L^-1^]	1.06 ± 0.46 (7)	1.92 ± 0.54 (2)	7.71 (1)
Particulate N [μg L^-1^]	35.68 ± 5.25 (7)	67.99 ± 2.39 (2)	89.45 (1)
Particulate organic C [μg L^-1^]	267.98 ± 37.94 (7)	441.97 ± 118.50 (2)	658.01 (1)
Dissolved organic C [μM]	121.79 ± 20.44 (6)	160.52 ± 39.31 (2)	141.71 (1)

Variables were measured constantly (water temperature; daily averages), weekly for several hours (pH, salinity, dissolved O_2_; sampling day average) or weekly in triplicate (nutrients and particulate as well as dissolved organic matter; sampling day average). The number of replicates for each parameter and season is displayed in brackets.

#### Physicochemical parameters

From May to November 2013, mean daily water temperatures in Bajo Rojo were on average 1.6 ± 0.1°C lower and more variable than in Matapalo. From December 2013 to April 2014, Bajo Rojo experienced on average 4.1 ± 0.2°C lower mean daily temperatures than Matapalo, and mean daily water temperatures dropped to a minimum of 16.0°C. To ensure that these temperature differences were not due to differences in water depth, temperature data from Matapalo were compared with data from a shallow reef (3 m water depth) close to Bajo Rojo. At this shallow reef, water temperature was still significantly lower than at Matapalo (U = 19836, *n* = 250, *p* < 0.001) and dropped to 18.4°C in upwelling season, thereby confirming a north-south gradient in upwelling intensity, independent of water depth. Mean daily water temperatures at Bajo Rojo and Matapalo were highly correlated to each other over the entire year (*r* = 0.877, *n* = 272, *p* < 0.001). During non-upwelling season, drops in temperature often occurred first at Bajo Rojo and were measured with a delay of about two days at Matapalo, whereas stronger upwelling events between February and April 2014 occurred simultaneously at both sites. Salinity at Bajo Rojo displayed the same temporal pattern than at Matapalo, with lowest values of 32.6 in December and an increase in upwelling season. Dissolved oxygen concentrations and pH at Bajo Rojo were on average lower than at Matapalo, with values ranging from 4.0 to 7.1 mg O_2_ L^-1^ and a pH of 7.75 to 8.10.

#### Inorganic nutrients and organic parameters

Similar to Matapalo, PO_4_^3-^ and NO_3_^-^ concentrations at Bajo Rojo peaked in February (1.2 ± 0.1 and 9.7 ± 0.4 μmol L^-1^ respectively) and were associated with highest chl *a* values (7.7 ± 0.3 μg L^-1^), whereas NH_4_^+^ concentrations were highest in April 2014 (3.0 ± 0.1 μmol L^-1^). Maximum PO_4_^3-^ and NH_4_^+^ concentrations were similar to Matapalo, while maximum concentrations of NO_3_^-^ and chl *a* were 1.4 and 3.5 times higher at Bajo Rojo. Particulate and dissolved organic matter concentrations at Bajo Rojo were in the same range than at Matapalo, experiencing similar temporal patterns with higher values during upwelling season (Table 2).

## Discussion

### Temporal variability of water parameters

The understanding of eastern tropical Pacific upwelling areas in terms of atmospheric forcing patterns, ocean physics and primary production has increased in the past three decades (reviewed by [13]), but dynamics of hydrological processes in coastal waters in general and coral reefs in particular remain understudied [12]. This study adds important insight into dynamics of inorganic and organic (dissolved and particulate) nutrients and biomass in the water column of upwelling influenced coral reefs. In the Gulf of Papagayo, changes in temperature and nutrient concentrations occurred in pulses during a major upwelling period from February to April 2014. Variability in physicochemical water parameters was more pronounced at the northern site confirming the proposed gradient in upwelling intensity, whereas parameters indicating sequential production and organic matter cycling were similar at both sites and remained elevated beyond the physical upwelling events.

#### Physicochemical parameters

Drops in seawater temperature of 2–9°C for several days indicated a pronounced influence of upwelling at the study sites, although the effect on temperature was smaller and shorter compared to coastal upwelling in the Gulf of Panama [12]. However, interannual variability in upwelling intensity is high and values comparable to Panama have been recorded in the Gulf of Papagayo in previous years [15]. The upwelling at the study sites increased surface salinities to over 34, thereby interrupting the low salinity zone beneath the Inter-Tropical Convergence Zone in the eastern tropical Pacific, where precipitation exceeds evaporation and salinities are usually below 33 [31]. Seasonal variability in salinity was therefore high, but still within the environmental limits for corals (25–42, [32]). Similar variability in salinity was observed in the Gulf of Panama (29.2–33.6, [12]). On a seasonal scale, dissolved oxygen concentrations were stable, whereas pH values were highest in February 2014 during strongest upwelling. In contrast, pH and oxygen concentrations in Papagayo Bay decreased during cold-water intrusions in a previous study [16]. Upwelling processes usually mix CO_2_-enriched and oxygen depleted deep waters into shallow water layers [33,34]. The high pH values recorded during upwelling in the present study may have been caused by increased primary productivity in the reef, as chl *a* concentrations during strongest upwelling events were up to 4-fold higher than average values. Oxygen concentrations however were not increased accordingly, suggesting that upwelling of low oxygenated water from the pronounced oxygen minimum layer below the tropical eastern Pacific [35] negated the positive effect of primary production on oxygen concentrations. We can however not exclude that increased pH values may have been an artifact of pH measurements. In contrast to previous studies where oxygen concentrations and pH were measured in the open water column, variability in water parameters very close to the reefs may reflect biological rather than transport processes. The observed semidiurnal ranges in oxygen and pH are similar to those in other tropical reef systems (1.7–3.0 mg O_2_ L^-1^ [36]; 0.10–0.25 pH units [37]) and are related to diurnal changes in biological processes such as photosynthesis and respiration [38,39]. The daily variability in oxygen and pH was high compared to seasonal variability, suggesting that metabolic processes such as photosynthesis and respiration are more important drivers for oxygen and carbonate chemistry variation directly on the reef than upwelling. Although minimum oxygen values in the early mornings were decreased during upwelling season, the reef organisms were able to buffer the upwelling induced changes in oxygen and pH which were detected in the open water column (e.g. observed by [16]).

#### Inorganic nutrients

In the present study, concentrations of inorganic nutrients in the reef water did not increase during months with high rainfall, indicating that precipitation and runoff were not important sources of nutrients. The delivery of new nutrients to the shallow reefs is therefore primarily controlled by seasonal upwelling. During non-upwelling and upwelling season, nutrient concentrations measured at Matapalo reef were in the range of average concentrations for most tropical reefs (0.25 ± 0.28 s.d. μmol NO_3_^-^ L^-1^, 0.13 ± 0.08 s.d. μmol PO_4_^3-^ L^-1^[1]). During strongest upwelling events, nitrate, phosphate and ammonia concentrations at Matapalo increased by 15-, 5- and 4-fold respectively. Maximum PO_4_^3-^ concentrations at Matapalo were similar to values measured in the Gulfs of Papagayo and Panama or in the Peruvian upwelling system, but maximum NO_3_^-^ concentrations were less than 50% (~15 μmol NO_3_^-^ L^-1^ [12,17,40]). As those studies measured nutrient concentrations in the open water column, lower concentrations on Matapalo reef suggest that the upwelling NO_3_^-^ was taken up quickly by pelagic and benthic primary producers. Comparing measured nutrient and temperature values during upwelling with water depth profiles from oceanographic stations in the Gulf of Papagayo [41], the properties of the upwelling water correspond to those of water from approximately 30–50 m water depth. This water layer represents the depth of the thermocline during non-upwelling season, and corresponds with thermocline depth in the Gulf of Panama [12]. At both locations, the thermocline shoals when strong offshore winds in dry season increase the surface flow and vertical mixing, delivering high concentrations of inorganic nutrients to the surface water [41].

#### Organic parameters

In several upwelling areas of the eastern tropical Pacific, high concentrations of nutrients stimulate primary productivity and phytoplankton growth [41]. Maximum chl *a* concentrations in the Gulf of Papagayo during upwelling were in the same range than average surface values for the highly productive Peruvian upwelling, indicating that the Papagayo upwelling seasonally supports productivity similar to one of the most productive upwelling regions of the world. However, annual average chl *a* concentrations in the seasonal upwelling areas of Papagayo, Tehuantepec and Panama are almost one magnitude lower than in the persistent Peruvian upwelling system (0.26–0.33 compared to 2.55 μg L^-1^ [41]). Peaks in chl *a* in the present study occurred asynchronous with peaks in nutrient concentrations. This suggests rapid uptake of available nutrients by primary producers, but a delay in biomass response as an initial rapid growth of phytoplankton after nutrient addition may take 3–5 days [42,43]. POC and PON increased together with chl *a* concentrations, illustrating the conversion of inorganic nutrients into organic matter. Very few studies in upwelling regions investigated organic parameters besides chl *a*, and even fewer studies measured these parameters directly above reef communities. There is no record from the eastern tropical Pacific, but comparable monitoring of particulate and dissolved organic matter on coral reefs was conducted in the Andaman Sea [44] and in Caribbean Colombia [45]. The increase in organic matter detected in the present study was much more pronounced than reported from those areas, where concentrations of particulate and dissolved organic matter were similar during non-upwelling, but did not increase significantly during upwelling [44,45]. Reef communities constantly take up inorganic nutrients, DOC and POM in particulate or remineralized form [46]. Suspended particles may therefore play a more significant role in reef nutrient cycling than previously considered [47]. We know of only few studies reporting similarly high pulses of organic matter and its implications on coral reefs. During coral spawning events in the Great Barrier Reef, PON increased 3- to 11-fold to maximum concentrations of 300 μg L^-1^, which resulted in a stimulation of oxygen consumption for one week and increased POM and chl *a* concentrations in the reef water for two weeks [48]. Nutrients released after coral spawning stimulated the autotrophic communities with 4.0- and 2.5-factor increases in pelagic and benthic production respectively for 4–5 days [49]. The spawning studies showed that even short term pulses of organic matter can influence the productivity of coral reefs and contribute substantially to reef nutrient budgets. In the Gulf of Papagayo, the effects of upwelling on chl *a* and organic matter lasted much longer compared to those studies, as all organic parameters remained elevated beyond the actual physical upwelling event indicated by temperature and nutrients. The accumulated input of nutrients and POM was therefore high, and 40–45% of the annual POC and PON production was supported within the three months of strongest upwelling. DOC in the investigated reefs already increased 2 weeks after the first upwelling event in December 2013 and stayed elevated throughout the upwelling season. The increase was likely caused by enhanced primary production and subsequent excess organic matter release by reef organisms such as algae and corals in response to increased nutrient availability [50,51]. The primary producer-derived DOC may affect the activity and growth of microbial communities in the reef, which play an important role in the remineralization of organic and inorganic matter [52] and transfer energy to higher trophic levels [53–56]. The fact that DOC stayed elevated for several months after the initial nutrient pulse indicates that the upwelling plays an important role in fueling the reef food web, particularly primary producers, at the study sites.

## Conclusions

This study describes temporal changes in a wide range of oceanographic parameters at a level of detail unprecedented in studies of eastern Pacific coral reefs. The observed effects of upwelling on temperature and nutrient concentrations confirm previous studies in the Gulf of Papagayo and other tropical upwelling regions, whereas the magnitude and temporal dynamics of cascading effects on chl *a* and organic matter concentrations have not been reported before. The results add important understanding of upwelling influences on coral reefs as they indicate that influences go beyond the effects of temperature or nutrient concentrations addressed in previous studies. Upwelling can be considered the key driver that controls productivity and nutrient recycling in coral reefs at the northern Pacific coast of Costa Rica. Local coral communities have to adapt to a high variability in water parameters, which likely influences functions and services of local coral reefs. Many reefs, not only in the eastern tropical Pacific, are influenced by regional-scale upwelling, e.g. located in the equatorial Pacific [57], in the Caribbean [45,58,59], in the Indian Ocean [60,61] and on the west coast of Australia [62]. Probably even more coral reef locations are influenced by large amplitude internal waves [44] or high frequency internal bores [63,64], which cause comparable changes in conditions in the scales of minutes to hours. The pronounced cascading effects of upwelling on organic matter cycles are possibly prevalent on most of these coral reefs, although regional differences are likely to occur. The quantification of inorganic as well as organic dissolved and particulate organic matter in future studies is essential to discuss the influence of upwelling on coral reef productivity and functioning and to evaluate the suitability of upwelling areas as refugee for global warming [65].
